# Simulation-based training in ear, nose and throat skills and emergencies

**DOI:** 10.1016/j.bjorl.2022.01.001

**Published:** 2022-02-10

**Authors:** Goutham MK, Marina Saldanha, Vadisha S. Bhat, Rajeshwary A, Mark Jittu Vincent, Aishwarya Ravikumar

**Affiliations:** Department of Otorhinolaryngology, Nitte (Deemed to be University), KS Hegde Medical Academy, Mangalore, Karnataka, India

**Keywords:** Medical education, ENT, Simulation, Mannequin training, Outcome-based

## Abstract

**Objectives:**

The aim of the study was to compare lecture-based teaching and simulation-based hybrid training for ENT induction and objectively assess the performance of trainees in a simulated environment.

**Methods:**

This is a prospective interventional study that included 60 interns in their rotatory internship with no prior exposure to ENT emergencies. The interns came in batches of 5‒6 for their 15-days ENT postings. On the first day, a pre-test questionnaire, lecture-based teaching on three scenarios and then allocation into one of the three simulation groups- Group A (Tracheostomy group), Group B (Nasogastric tube group), and Group C (Epistaxis group) was done. Hands-on simulation training was given only to the assigned group. At the end of 15-days, post-test questionnaire and an objective assessment of the three scenarios in a simulated environment was conducted. The same training was repeated for each batch of participants who attended the posting.

**Results:**

The participants had significant improvement in the post-test scores in all three scenarios (*p* <  0.05), and these improvements were marked in those who had received simulated training. On comparing simulation scores, the participants who received hands-on training on a particular scenario outperformed other (*p* <  0.05).

**Conclusion:**

Simulation-based training improves cognition and overall confidence in managing ENT skills and emergencies. In simulation training, objective and standardized assessment is the key to achieve specific learning objectives to improve the psychomotor and cognitive skill.

**Level of evidence:**

II.

## Highlights

• Simulation in ENT training develops cognition, psychomotor skills and confidence.
• Hybrid teaching method enhances the knowledge and psychomotor skills.
• Simulation training followed by objective assessment improves retention and application.

## Introduction

ENT (Ear, Nose and Throat) emergencies are not uncommon and can often be life-threatening without emergency management. In casualty and emergency departments, junior doctors are the first-on-call responders rather than the senior consultants.^1^ Unlike the knowledge of the disease, the confidence of interns in managing ENT emergencies may not always be up to the mark and this might give rise to patient safety issues when it comes to emergency cover. In such instances, adequate knowledge, confidence and skilled management is required to manage the emergency and reduce complications.

The practice of learning skills by trainees on any patient raises concerns about patient safety.^2^ Simulation-based education being added to the medical curriculum has mitigated these situations and has shown to be a practical method of teaching both individual procedures^3^ and systematic care.^4^ Use of simulation is widespread in teaching laparoscopic surgery, endoscopy, emergency resuscitation, essential clinical skill and team leadership.^5^

The advantage of medical skills simulators is that it can be used both as training devices as well as an assessment tool.^6^ Although high fidelity simulators are an added advantage for medical simulation, standardized surgical and non-surgical tasks can be performed on low fidelity simulators.

Our aim of the study was to compare lecture-based teaching and simulation-based hybrid approach in the management of ENT clinical skills and emergencies and highlight the importance of assessment after simulation training.

## Methods

This was a prospective interventional study conducted in Department of Otorhinolaryngology. Ethical clearance was taken from Institutional Ethics Committee (INST.EC/EC/068/2019-20) and permission from the simulation laboratory. The study was conducted over a seven-month period from June 2019 to December 2019.

### Participants

The participants were interns, who had completed their four-year undergraduate medical program and had their first entry to Otorhinolaryngology postings. They joined the ENT department in batches of 5‒6 every 15-days as a part of compulsory rotatory internship. Every 15-days the study program was repeated for all batches. Written informed consent was taken and all the participants who completed pre-test, post-test and simulation assessment were included in the study. Based on statistical analysis, our study needed a total of 60 students, with 20 students in each simulation group for comparison of scores.

### Study design (Fig. 1)

Three clinical scenarios were selected in Otorhinolaryngology set up-tracheostomy tube care, anterior epistaxis management and nasogastric (Ryle’s) tube insertion.

**Figure 1:**
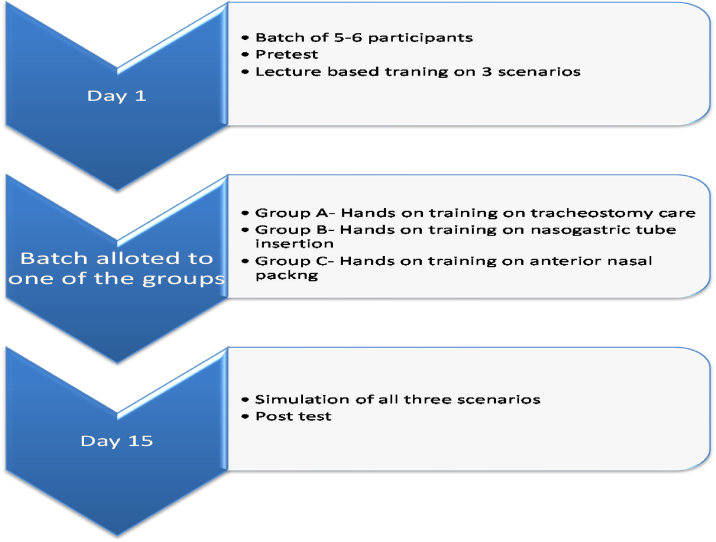
Participants journey.

On day 1, the participant batch of 5‒6 interns received a pre-test with a validated questionnaire on three scenarios. The test was conducted without any prior warning to the participants, in an examination environment. The questionnaire included Multiple-Choice Questions (MCQ) and questions assessing knowledge, attitude and confidence in managing these scenarios with modified Likert scale. Following which a 1-hour lecture-based teaching program by an ENT consultant was taken on the three scenarios. The lecture included a PowerPoint presentation of clinical management and performance of the clinical skill with video demonstration.

**Figure 2:**
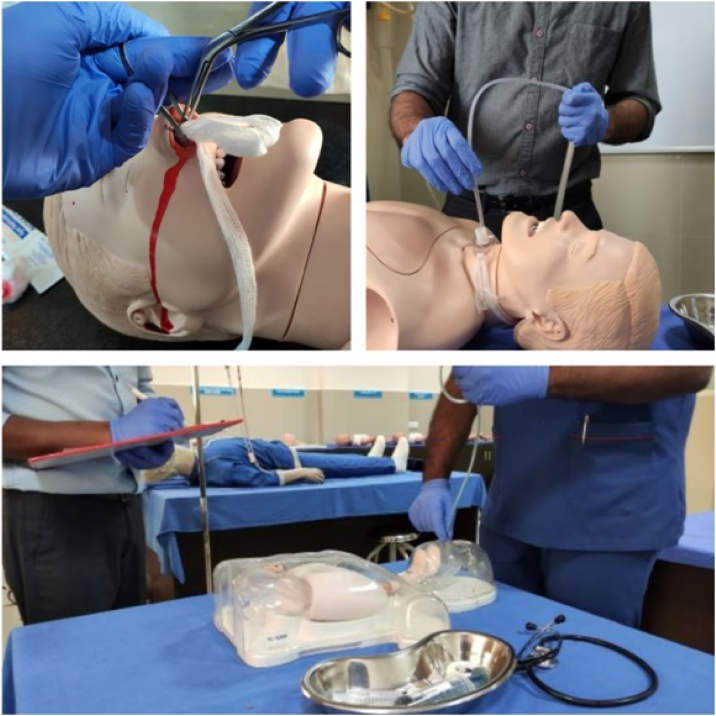
Demonstration of anterior nasal packing, tracheostomy tube care and assessment of nasogastric tube insertion with checklist by examiner.

Later the participants were allotted to Group A (Tracheostomy group), Group B (Nasogastric tube group) or Group C (Epistaxis group) according to their entry to ENT postings. The first batch of interns was allotted to Group A, second batch to Group B, third batch to Group C and similar allocation was followed with subsequent batches. Each group received simulation based hands-on training on mannequins (NW8 Tube Feeding Simulator [NG, OG AND PEG] Kyoto Kagaku Co., Ltd, and NG tube and trach care trainer 375‒10001 Laedral medical) on the assigned scenario. Group A received training on tracheostomy tube care, Group B on Nasogastric Tube (NG) insertion, and Group C on managing epistaxis (Fig. 2).

### Assessment

At the end of 2-weeks, the participants had to demonstrate the management of all the three situations on mannequins under supervision of an ENT consultant. Their performance was assessed with the help of a checklist, thus standardizing the scoring. Three assessors were involved during the post-test simulation-based hands-on assessment to minimize researcher bias. After the candidates had completed demonstration, they received a post-test with same questionnaire as before. Structured feedback was collected at the end of the evaluation and all students received simulation based hands-on training on the other 2 scenarios as well.

### Pre-test & post-test validity

The questionnaire was designed by the authors aligning with the objectives of the study. It included modified Likert scale type questions, Multiple Choice Questions and Checklist type of questions to assess the confidence and knowledge of the participants. The questions were validated by a panel of 3 experts, both within and outside the university and were not involved in the study. They evaluated the accuracy and the adequacy of the response options. Both pre and post-test used the same questionnaire and were practical to the concepts taught in the program. The checklist for the evaluation of participants clinical skills were also validated by the same group of experts.

### Data collection and analysis

All pre-test and post-test questionnaire assessment responses and simulated demonstration scores were entered separately for each group. Only those participants who had completed both the pre-test and the post-test were included in the analysis. The data were analysed with the IBM SPSS statistics program version 20.0 (IBM, Armonk, NY, USA). Paired *t-*test was used to compare the mean scores in the pre- and post-test questionnaire. One-way analysis of variance (ANOVA) test and post-hoc Tukey test was used for comparing the three groups.

## Results

60 interns participated in the study. Each simulation group (Tracheostomy care, epistaxis management and Nasogastric tube insertion) had 20 participants each.

### A. Pre-test questionnaire scores

Comparison of pre-test scores was done on all three scenarios and no significant difference was observed between the three groups as none of the participants had received any form of training on the three scenarios before taking the pre-test.

### B. Post-test questionnaire scores (Table 1)

Group A (Tracheostomy group) showed statistically significant improvement in post-test scores on epistaxis management, tracheostomy care and nasogastric tube care (*p* =  0.001, 0.001 and 0.018 respectively). Group B (NG tube group) and Group C (Epistaxis group) also showed similar trend in post-test scores. All groups showed significant improvements in the post-test scores regardless of their training method.

**Table 1:** Comparison of pre and post test scores between three groups.

Group (N = 60)	Mean ± SD	Mean difference ± SD	*p*-value
Group A (Trachesotomy Group) n = 20			
Pre-test Epistaxis Mx	15.6 ± 3.42	-7.15 ± 3.5	< 0.001
Post-test Epistaxis Mx	22.75 ± 3.09		
Pre-test Trach tube care	12.15 ± 4.44	-5.6 ± 3.93	< 0.001
Post-test Trach tube care	17.75 ± 1.74		
Pre-test NG tube care	19.4 ± 4.33	-2.5 ± 4.31	0.018
Post-test NG tube care	21.9 ± 2.85		
Group B (NG tube Group) n = 20			
Pre-test Epistaxis Mx	15.35 ± 2.18	-5.95 ± 3.09	<0.001
Post-test Epistaxis Mx	21.3 ± 2.81		
Pre-test Trach tube care	12.05 ± 3.61	-2.9 ± 3.73	0.003
Post-test Trach tube care	14.95 ± 3.58		
Pre-test NG tube care	19.15 ± 4.22	-3.65 ± 4.56	0.002
Post-test NG tube care	22.8 ± 2.33		
Group C (Epistaxis Group) n = 20			
Pre-test Epistaxis Mx	14.9 ± 2.9	-9.4 ± 3.24	< 0.001
Post-test Epistaxis MX	24.3 ± 2.13		
Pre-test Trach tube care	11.4 ± 2.35	-3.6 ± 2.64	< 0.001
Post-test Trach tube care	15 ± 2.53		
Pre-test NG tube care	18.95 ± 4.81	-2.25 ± 3.46	0.009
Post-test NG tube care	21.2 ± 3.96		

SD, Standard Deviation; Mx, Management; NG, Nasogastric; Trach, Tracheostomy.

Table 1 shows comparison of pre-test and post-test scores in knowledge and confidence in management of epistaxis, Nasogastric tube insertion and Tracheostomy care. All participants scored better in post-test (*p* <  0.05).

### C. Comparison of post-test scores between the three groups using one way ANOVA test and post-hoc Tukey test (Table 2)

#### a) Comparison of post-test scores of epistaxis management

Group C (Epistaxis group) had the highest mean value of 24.3 and Group B (NG tube group) had the least value of 21.3. This difference is statistically significant (*p* = 0.004) favouring Group C. This is confirmed by post-hoc Tukey test, where comparing Group B with Group C shows statistically significant difference (*p* = 0.003). Comparison of Group A with Group C did not show any statistical significance.

**Table 2:** Comparison of the evaluation scores (post-test and simulation) between three groups.

	Group A Tracheostomy (n = 20)	Group B Nasogastric tube (n = 20)	Group C Epistaxis (n = 20)	One way ANOVA	Post-hoc Tukey test		
				*p*-value	Group A vs Group B difference (*p*-value)	Group A vs Group C difference (*p*-value)	Group B vs Group C difference (*p*-value)
**Post-test Epistaxis Mx**	22.75 ± 3.09	21.3 ± 2.81	24.3 ± 2.13	**0.004**	1.45 (0.217)	-1.55 (0.176)	**-3 (0.003)**
**Post-test NG tube care**	21.9 ± 2.85	22.8 ± 2.33	21.2 ± 3.96	0.274	-0.9 (0.635)	0.7 (0.759)	1.6 (0.244)
**Post-test Trach. Tube care**	17.75 ± 1.74	14.95 ± 3.58	15 ± 2.53	**0.002**	**2.8 (0.005)**	**2.75 (0.006)**	-0.05 (0.998)
**Improvement Epistaxis Mx**	7.15 ± 3.5	5.95 ± 3.09	9.4 ± 3.24	**0.005**	1.2 (0.483)	-2.25 (0.085)	**-3.45 (0.004)**
**Improvement in Trach tube care**	5.6 ± 3.93	2.9 ± 3.73	3.6 ± 2.64	**0.046**	**2.7 (0.045)**	2 (0.173)	-0.7 (0.801)
**Improvement NG tube care**	2.5 ± 4.31	3.65 ± 4.56	2.25 ± 3.46	0.525	-1.15 (0.656)	0.25 (0.98)	1.4 (0.536)
**Simulation Nasal packing**	3.45 ± 0.95	3.55 ± 1.23	4.8 ± 0.83	**<0.001**	-0.1 (0.948)	**-1.35 (<0.001)**	-**1.25 (0.001)**
**Simulation Trach. Tube care**	5.6 ± 1.43	4.15 ± 1.31	4.6 ± 2.01	**0.019**	**1.45 (0.017)**	1 (0.131)	-0.45(0.653)
**Simulation NG tube insertion**	4.45 ± 0.69	5.65 ± 0.88	5.75 ± 0.91	**<0.001**	**-1.2 (<0.001)**	**-1.3 (<0.001)**	-0.1 (0.923)

Gp, Group; Mx, Management; NG, Nasogastric; Trach, Tracheostomy.

Table 2 shows evaluation of students on 15^th^day. Participants who had received hands on training in nasal packing and tracheostomy tube care skills showed statistically significant improvement in their performance during their evaluation in simulated environment.

There was no significant difference in the scores between Group A and Group B. This can be explained by the fact that both these groups did not receive additional simulation-based training on epistaxis management and nasal packing.

#### b) Comparison of post-test scores of tracheostomy tube care

Group A (Tracheostomy group) had statistically significant scores (*p* = 0.002). Post-hoc Tukey test comparing the three groups also showed that tracheostomy group outperforms the other groups.

#### c) comparison of post-test nasogastric tube care

There was no statistical significance between the three groups.

Nasogastric tube insertion being a common procedure, the participants may have had prior experience with this procedure in their previous postings in other departments and may explain the above results.

#### d) Comparison of simulation scores

Group C had highest simulation scores in nasal packing for Epistaxis (*p* = 0.001). The participants who received hands-on simulation training in nasal packing outperformed others when it came to practical demonstration.

**Figure 3:**
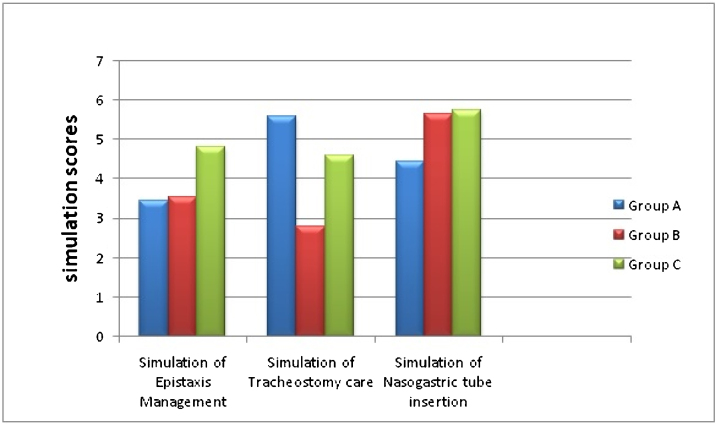
Simulation scores comparing the lecture-based and simulation-hybrid training in each scenario.

Simulation in Tracheostomy tube suctioning showed statistically significant values for Group A (*p* =  0.019). This significance was present only while comparing with Group B and not with Group C (Fig. 3).

Simulation in Nasogastric tube insertion scenario showed significant scores (*p* < 0.05) in both Group B and Group C on comparing with Group A.

### D. Confidence levels among participants

**Figure 4:**
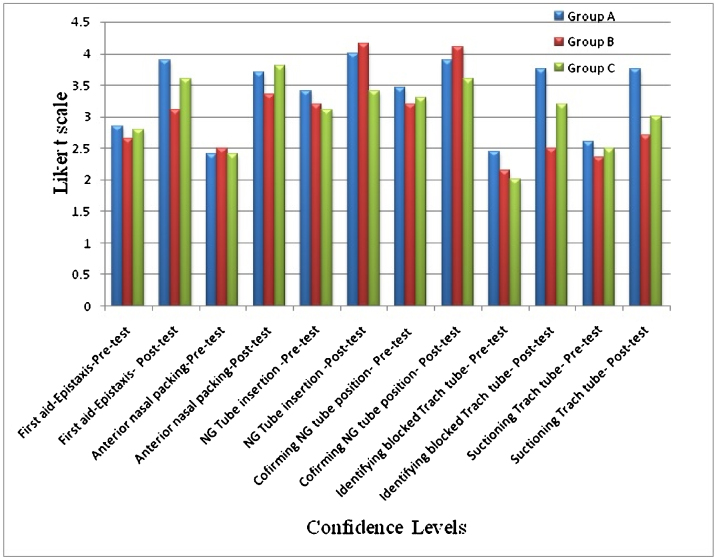
Confidence level of participants in ENT scenarios.

The participants had better scores in their confidence levels at the end of the program and these confidence levels were more in the groups which received simulation training (Fig. 4).

## Discussion

With the advancements in medical education, simulation-based training has become essential and has even been included as a part of medical curriculum in the form of Competency Based Medical Education (CBME).^7^, ^8^

In this study, we assessed the knowledge, confidence, experience and skill of our participants with regards to ENT emergencies and procedures. Kirkpatrick Level 2 model^9^ was used for analyzing and evaluating the results of the training. The participant’s performance in lecture-based teaching and simulation-based hybrid training was compared in common ENT scenarios-epistaxis management, tracheostomy tube care and nasogastric tube insertion. We further measured the competence in performing the skill by a validated checklist and questionnaire.

Based on our pre-test scores, we acquired base-line information about their knowledge, confidence and skill in performing the three scenarios. With this information, we assessed their improvement during this training program. There was significant improvement in the post-test scores in all three scenarios even without simulation-based training (*p* < 0.05). On further analysis of the results, the improvement in the post-test scores of simulation training was significant in most groups compared to lecture-based training alone. The Likert scale grading showed that the confidence levels in performing these tasks were higher in the participants who received simulation- based training. This evidence shows that simulation-based hybrid approach of training improves the cognitive and psychomotor skills along with overall confidence. A review of other programs which assessed skills and confidence after simulation had similar results.^10^, ^11^ The concept of organizing boot camps at the beginning of internship also improves overall confidence and performance.^12^

The key components of a successful simulation program have been identified as outcome measurement, skill acquisition, curriculum integration and educational/professional context.^13^ Assessment of the skills in a simulated environment showed that participants who received simulation-based training in a particular scenario outdid the participants who were not trained in simulation. Our outcome–based evaluation method helped us to objectively validate the assessment and identify potential areas for participant’s improvement. This has been validated in other studies, where they had specific outcome measurement tools.^11^, ^14^

### Limitations

The participants during their postings in ENT department might have been exposed to these scenarios and this could have lead to bias during assessment. Secondly, the interns were assessed only once on the 15^th^day from initial training. Repeat assessment after three months would have further validated the study, but since the participants had postings in other departments, our assessment had to be completed within this time frame of 15-days.

## Conclusion

Simulation helps in improving the cognitive, psychomotor and confidence levels of the participants. Objective structured assessment is the key to achieve specific learning objectives in simulation training.

## Funding

This research received no specific grant from any funding agency in the public, commercial, or not-for-profit sectors.

## Conflicts of interest

The authors declare no conflicts of interest.

## References

[1] Smith M.E., Navaratnam A., Jablenska L., Dimitriadis P.A., Sharma R. (2015). A randomized controlled trial of simulation-based training for ear, nose, and throat emergencies. Laryngoscope.

[2] Rosenthal R., Gantert W.A., Hamel C., Metzger J., Kocher T., Vogelbach P. (2008). The future of patient safety: surgical trainees accept virtual reality as a new training tool. Patient Saf Surg.

[3] Sturm L.P., Windsor J.A., Cosman P.H., Cregan P., Hewett P.J., Maddern G.J. (2008). A systematic review of skills transfer after surgical simulation training. Ann Surg.

[4] Cook D.A., Brydges R., Hamstra S.J., Zendejas B., Szostek J.H., Wang A.T. (2012). Comparative effectiveness of technology enhanced simulation versus other instructional methods: a systematic review and meta-analysis. Simul Healthc.

[5] Cook D.A., Hatala R., Brydges R., Zendejas B., Szostek J.H., Wand A.T. (2011). Technology-enhanced simulation for health professions education: a systematic review and meta-analysis. JAMA.

[6] Tavakol M., Mohagheghi M.A., Dennick R. (2008). Assessing the skills of surgical residents using simulation. J Surg Educ.

[7] Shah N., Desai C., Jorwekar G., Badyal D., Singh T. (2016). Competency-based medical education: An overview and application in pharmacology. Indian J Pharmacol.

[8] Sagi D., Pessach-Gelblum L., Divon-Ophir O., Rubinstein R., Laufer S., Sela R. (2019). Simulation as a training and assessment tool for competency based medical education – A regulatory challenge. Harefuah..

[9] La Duke P. (2017). How to evaluate training: using the kirkpatrick model. Prof Saf.

[10] Bhalla S., Beegun I., Awad Z., Tolley N. (2020). Simulation-based ENT induction: validation of a novel mannequin training model. J Laryngol Otol.

[11] Kumar A., Nestel D., East C., Hay M., Lichwark I., McLelland G. (2018). Embedding assessment in a simulation skills training program for medical and midwifery students: A pre- and post-intervention evaluation. Aust N Z J Obstet Gynaecol.

[12] Malekzadeh S., Malloy K.M., Chu E.E., Tompkins J., Battista A., Deutsch E.S. (2011). ORL emergencies boot camp: using simulation to onboard residents. Laryngoscope.

[13] McGaghie W.C., Issenberg S.B., Barsuk J.H., Wayne D.B. (2014). A critical review of simulation-based mastery learning with translational out-comes. Med Educ.

[14] Mikasa A.W., Cicero T.F., Adamson K.A. (2013). Outcome-based evaluation tool to evaluate student performance in high-fidelity simulation. Clin Simul Nurs.

